# Aggression and emotional distress in adolescents: a cross-sectional chain mediation model of internet addiction and somatization

**DOI:** 10.3389/fpsyt.2026.1828955

**Published:** 2026-05-26

**Authors:** Zhewei Su, Yiting Kong, Yuancen Zhong, Rui Wang, Qi Zhang, Jianmei Chen, Su Hong, Li Kuang

**Affiliations:** 1 Psychiatric Center, The First Affiliated Hospital of Chongqing Medical University, Chongqing, China; 2 Department of Psychiatry, The First Affiliated Hospital of Chongqing Medical University, Chongqing, China

**Keywords:** adolescents, aggression, anxiety symptoms, chain mediation, depressive symptoms, internet addiction, somatization

## Abstract

**Background:**

Adolescent depression and anxiety are major public health concerns. Aggression is frequently associated with internalizing symptoms, but the behavioral and body related mechanisms underlying this association remain insufficiently clarified. This study examined a theoretically proposed chain mediation model linking aggression with depressive and anxiety symptoms through internet addiction and somatization.

**Methods:**

A cross-sectional survey was conducted among 5,307 high school students in Chongqing, China. Participants filled in the Buss and Perry Aggression Questionnaire (BPAQ), Internet Addiction Test (IAT), Patient Health Questionnaire - 15 (PHQ - 15), Patient Health Questionnaire - 9 (PHQ - 9) and Generalized Anxiety Disorder Scale - 7 (GAD - 7). Regression based chain mediation analyses with 5,000 bootstrap samples were performed using PROCESS Model 6, with gender and age controlled as covariates.

**Results:**

The results showed that aggression was positively correlated with depressive symptoms (β = 0.256, p < 0.001) and anxiety symptoms (β = 0.275, p < 0.001). Chain mediation analysis showed that aggression was indirectly associated with mental health through three distinct pathways: 1. the independent mediating effect of internet addiction; 2. the independent mediating effect of somatization; 3. the sequential chain mediating effect from internet addiction to somatization. The model explained more variance in depressive symptoms (R² = 58.2%) than in anxiety symptoms (R² = 53.5%). Furthermore, the association between somatization and depressive symptoms (β = 0.457) was stronger than that between somatization and anxiety symptoms (β = 0.428).

**Conclusion:**

This study supports a statistically significant chain mediation pattern in which aggression is associated with depressive and anxiety symptoms through internet addiction and somatization. The findings suggest that somatization may represent an important body related correlate in the association between maladaptive digital behavior and emotional distress, with a slightly stronger association observed for depressive symptoms than for anxiety symptoms. These findings highlight the importance of integrated school based interventions that address digital behavior regulation, somatic symptom monitoring, and emotional distress among adolescents with higher aggressive tendencies.

## 1 Introduction

Mental health among adolescents in the 21st century is a global public health issue (1). Adolescence is a transformative stage characterized by neurobiological changes, identity formation, and social environmental shifts (2). At this stage, the prefrontal cortex is not fully mature, and top-down control is not yet fully developed, so adolescents are more sensitive to emotional stimuli (3). The asynchronous development of the limbic system and prefrontal cortex in adolescence makes them more prone to internalizing disorders such as depression and anxiety (4). This vulnerability is reflected in the global health burden, where mental health accounts for 15% of the disease and injury burden among people aged 10-19, and depression is the leading cause (1). In China, the rapid social changes and heavy academic pressure make the risk more serious (5). Epidemiological studies have found that nearly a quarter of Chinese middle school students have persistent depressive or anxiety symptoms, which are related to serious consequences such as self-harm, academic decline, and long-term psychosocial impairment (6–8).

Research on the pathology of internalizing problems has gradually focused on aggression, which is traditionally classified as an “externalizing behavior” (9–11). Aggression includes physical violence, verbal hostility, and anger experiences, it is not only a form of overt resistance but also often a maladaptive emotion regulation strategy (12). Interpersonal theories of depression suggest that adolescents with high trait aggression are socially isolated and excluded by peers due to interpersonal conflicts, a process termed “social erosion” (13–17). The outcome of social erosion—namely, a lack of stable social support—acts as a potent stressor that may contribute to psychological states such as despair and self-deprecation, ultimately leading to depression and anxiety disorders (14). The link between aggressive behavior and subsequent emotional disorders has been supported, but there are key gaps that need to be understood. How aggressive traits are transformed into clinical symptoms through specific behavioral and physiological paths has not been fully elucidated.

Internet addiction is the critical factor mediating the proposed pathway at the behavioral level. As digital technology is ubiquitous in adolescents’ daily lives, internet addiction has emerged as a common maladaptive coping strategy (18, 19). The Social Compensation Hypothesis provides an explanation. The hypothesis suggests that aggressive teenagers have difficulty processing real social cues, and when encountering peer rejection, they withdraw into the cyberspace to seek dominance or emotional comfort (20, 21). This “digital escape” often turns into compulsive use, manifested as loss of control over internet use and neglect of offline responsibilities (22). Previous studies also show that internet addiction can disrupt the brain’s reward circuit and natural circadian rhythms (23, 24). These physiological consequences are core factors in the etiology of emotional disorders (25, 26).

In particular, the impact of internet addiction is not merely psychological but may become somatized (27). Somatization is a clinical description of psychological stress manifested by physical symptoms such as chronic fatigue, persistent headaches, or gastrointestinal distress even when there is no clear organic pathology (28). This is an effective link between somatic and affective states. In the Biopsychosocial Model, the mind and body represent a single system (29), and in this system, chronic behavioral stress (such as stress from internet addiction) may result in persistent sympathetic nervous system hyperarousal (30). This global pathological state can then lead to somatic symptoms (31). The link between somatic states and affective states is quite prominent in adolescents. This link is explained by developmental factors: since an adolescent’s ability to express emotions verbally is still developing, the physical body often actively expresses what the mind experiences (32). Therefore, somatic complaints in this context should not be dismissed as mere incidental “side effects”; rather, they function as physiological potential early indicators. These potential early indicators signal a stress-response system that has become overburdened, and they hold the potential to either contribute to the onset of or exacerbate existing overt psychiatric conditions (33, 34).

A chain mediation model linking aggression to mental health outcomes through the sequential roles of internet addiction and somatization appears theoretically plausible. However, significant gaps continue to exist within the current body of literature that must be acknowledged. The first gap concerns the integration of different types of mediators (i.e., behavioral and physiological). More specifically, while individual pairwise links (e.g., aggression to internet addiction, or internet addiction to depression) are each documented quite extensively in research, few have integrated both a behavioral mediator (internet addiction) and a physiological mediator (somatization) into a single, unified chain mediation framework. The second gap is the distinction between specific mental health outcomes. Although depressive and anxiety symptoms are frequently co-occurring, they are characterized by quite different symptoms. For example, depressive symptoms often comprise “low energy” of behavior and withdrawal from activities, anxiety symptoms are often “hyperarousal” (i.e. physiological alertness) (35). Considering these differences, there are problems that have not been solved: it is not clear whether the proposed somatization path has the same impact on the two results. The final gap concerns the statistical power of existing research. Most studies use relatively small sample sizes, and small samples may lack statistical power. This limitation is relatively crucial. In the chain mediation model, precise estimation of complex indirect effects requires strong analytical ability, but such ability is not available in the case of small samples.

**Figure 1:**
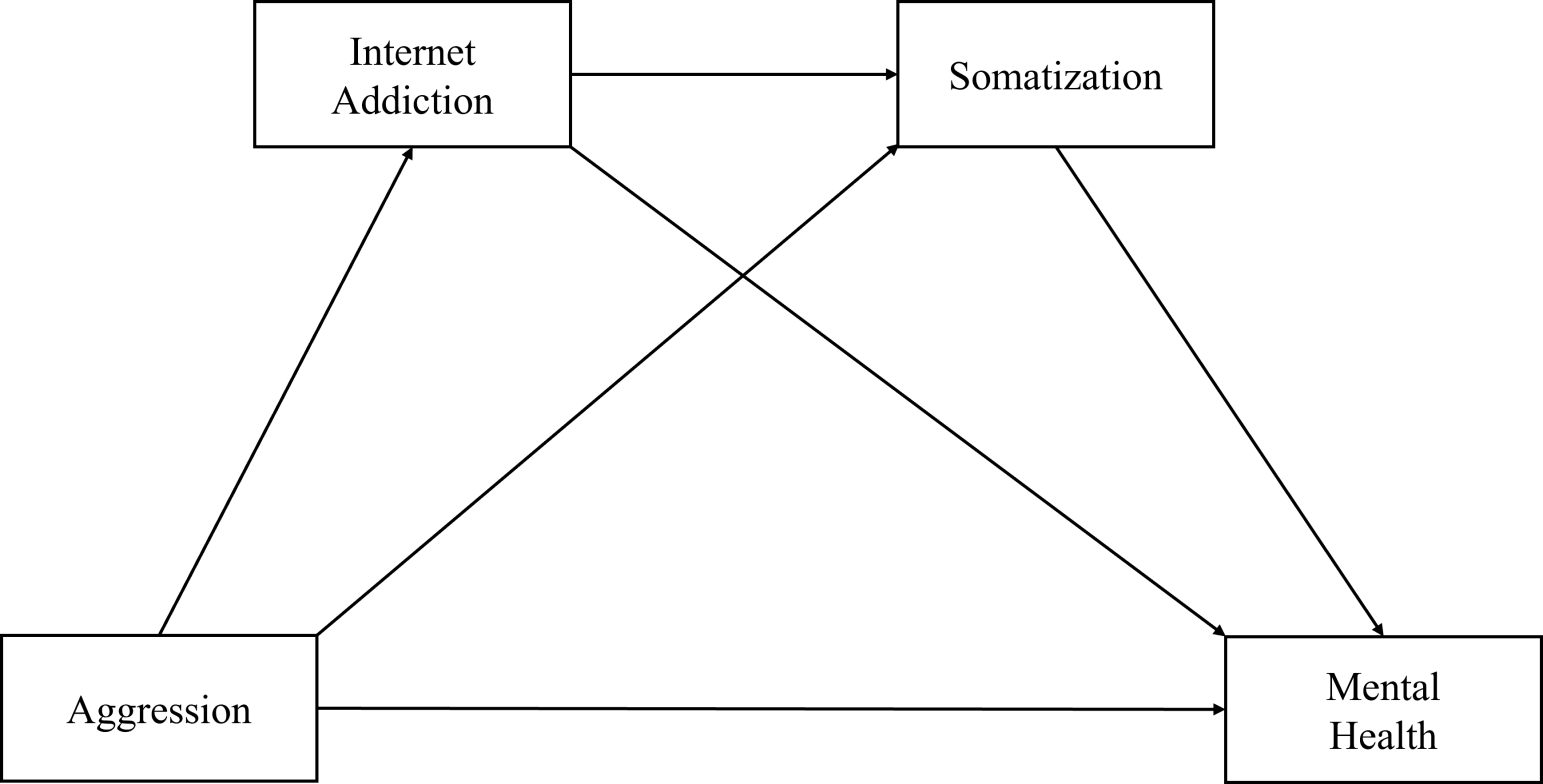
Hypothetical chain mediation model linking aggression to mental health (depressive/anxiety symptoms) via internet addiction and somatization.

To address the above limitations, the present study was conducted. A stable and accurate data set including 5,307 Chinese adolescents was used, and the method was based on dimensional symptoms. The contribution of the present study lies not in proposing entirely new mechanisms, but in integrating the established mechanisms into a unified comparative model and examining whether the same mediation pattern applies similarly to depressive and anxiety symptoms in a large adolescent sample. In this case, there are three objectives in this study: 1. To test the chain mediating effect of internet addiction and somatization in the relationship between aggression and mental health (**Figure 1**); 2. Compare the structural paths of depressive and anxiety symptoms; 3. Provide a basis for future specific interventions, particularly those implemented within school settings.

With a large sample of Chinese adolescents, this study provides statistical evidence for a theoretically proposed “trait-behavior-physiology-psychology” chain mediation framework. However, given the cross-sectional design, the model should be interpreted as a pattern of associations rather than a confirmed developmental trajectory. Finally, this work seeks to provide greater insight into adolescent psychopathology as it manifests in the current digital age.

## 2 Materials and methods

### 2.1 Participants and procedure

This study employed a cross-sectional design. Participants were recruited from several high schools in the Shapingba District of Chongqing, China. As a major municipality in Southwest China, Chongqing represents a highly urbanized region with an educational intensity and digital penetration rate consistent with many Tier-1 and Tier-2 Chinese cities. While the findings are representative of this specific urban-industrial context, they provide a valuable baseline for understanding the psychological dynamics of Chinese adolescents in modern metropolitan environments. Participants were recruited via a cluster sampling technique. Before data collection, informed consent was obtained from each student and their legal guardians. The survey was administered during regular class time. And the survey was done by research assistants who had received appropriate training. The assistants instructed all students that the study was totally voluntary and always anonymous. In addition, the assistants told the students that they could quit at any time without negative consequences.

Initially, 5,423 student questionnaires were collected. To ensure data completeness and quality, a multi-step screening process was applied, excluding: 1) questionnaires with missing data; 2) questionnaires with obvious response patterns (e.g., identical answers across all items). Using the exclusion criteria, some initial surveys were excluded from the data set. The remaining 5,307 questionnaires were valid and served as the final sample for testing. The effective response rate was 97.86%.

### 2.2 Measures

#### 2.2.1 Buss and Perry aggression questionnaire

In this study, Buss and Perry Aggression Questionnaire (BPAQ) was used to evaluate aggressive traits (36). The version utilized was the Chinese adaptation of this questionnaire. This adaptation had been previously validated for linguistic and cultural relevance in a study by Li et al. (37). The Chinese version BPAQ consists of 30 items across five dimensions: physical aggression, verbal aggression, anger, hostility and self-directed aggression. Items are rated on a 5-point Likert scale ranging from 1 to 5 (not at all like me to completely like me) (37). Higher scores can reflect higher aggressive traits levels. In the present study, the Cronbach’s alpha for the BPAQ was 0.937. While the BPAQ measures physical and verbal aggression, anger, and hostility, the current study utilized the total score to reflect a global trait of ‘aggressive tendency.’ This holistic approach allows for a generalized understanding of how an aggressive personality profile contributes to the behavioral-physiological chain, although future research might benefit from exploring the differential impact of specific dimensions.

#### 2.2.2 Internet addiction test

The severity of pathological internet use was measured using Internet Addiction Test (IAT) (38). The Chinese version of the IAT was validated (39). This 20-item scale evaluates the degree to which internet use interferes with an individual’s daily functioning, social life, and sleep patterns. Each item is scored on a Likert scale (5-point, 1 = rarely, 5 = always). Total scores of this test range from 20 to 100, with higher scores indicating more severe IA. The Cronbach’s alpha for the IAT in this sample was 0.931.

#### 2.2.3 Patient health questionnaire-15

The severity of somatic symptoms was evaluated using the Patient Health Questionnaire-15 (PHQ-15) (28). The Chinese version of the PHQ-15 had been validated as a reliable screening tool for somatization in Chinese populations (40). Participants were asked to rate how much they were troubled by 15 common physical symptoms (e.g., stomach pain, headaches, dizziness) during the past 4 weeks on a 3-point scale (0 = not troubled at all, 2 = troubled a lot). Because sexual activity is an extremely sensitive and private topic among Chinese middle school students, schools do not want us to inquire about it, so the item related to sexual activity was excluded. In this study, the Cronbach’s alpha for the PHQ-15 was 0.908.

#### 2.2.4 Patient health questionnaire-9

Depressive symptoms were assessed by using the Patient Health Questionnaire-9 (PHQ-9) (41). The PHQ-9 is a widely validated 9-item tool based on DSM-IV criteria for depression. The reliability and validity of the Chinese version PHQ-9 have been well-documented (42). The 9-item scale assesses the frequency of depressive symptoms over the past 2 weeks based on DSM-IV, with items scored from 0 to 3 (not at all to nearly every day). The total score of this questionnaire ranges from 0 to 27, where scores ≥ 10 are typically used as a cutoff for moderate depressive symptoms. The Cronbach’s alpha for the PHQ-9 in this study was 0.899.

#### 2.2.5 Generalized anxiety disorder-7

The Generalized Anxiety Disorder-7 (GAD-7) scale was used to measure the anxiety symptoms’ severity (43). The Chinese version of the GAD-7 had shown high sensitivity and specificity in Chinese (44). It consists of 7 items rated on a 4-point Likert scale (0 = not at all, 3 = nearly every day). Higher scores can indicate higher levels of anxiety. In the present study, the Cronbach’s alpha of 0.931 for the GAD-7 demonstrated strong internal consistency.

### 2.3 Statistical analysis

Data analysis was performed using IBM SPSS Statistics version 26.0. Given that all data in this study were collected through self-report measures, common method bias (CMB) could potentially influence the validity of the findings, so Harman’s single-factor test was conducted first (45). Second, descriptive statistics were conducted on demographic variables and the primary psychological measures. Third, Pearson correlation analysis was employed to examine the bivariate relationships among aggression, IA, somatization, and internalizing symptoms (depressive symptoms and anxiety symptoms). Finally, multiple linear regression analysis was conducted to test the direct associations within the model.

To mitigate potential multicollinearity, all primary continuous variables were standardized (Z-scores) before the mediation analysis. The chain mediation model was tested using the PROCESS macro for SPSS (Model 6) (46). In the model, the independent variable was aggression (X), depressive symptoms (Y_1_) and anxiety symptoms (Y_2_) were the dependent variables, and internet addiction (M_1_) and somatization (M_2_) were sequential mediators. Gender and age were included as covariates because they were available in the current dataset and are commonly associated with adolescent emotional and somatic symptoms. Other potentially important contextual variables, such as socioeconomic status, family environment, academic stress, and prior mental health history, were not available and were therefore discussed as limitations.

To evaluate the significance of the indirect effect in the mediation model, the bias-corrected bootstrap method was used. 5000 bootstrap resamples were generated from the original data. If the 95% confidence interval (CI) did not contain 0, then the indirect effect is statistically significant.

## 3 Results

### 3.1 Common method bias test

To test potential common method bias (CMB) in self-reported data, the Harman single-factor test was performed at first (45). The first unrotated factor explained 34.534% of the total variance, which was below the commonly used threshold of 40%. This result suggests that no single factor dominated the covariance among the variables. However, Harman’s single factor test cannot completely rule out common method bias, and the possibility of shared method variance should still be considered when interpreting the findings.

### 3.2 Demographic characteristics

This study finally involved 5,307 participants for analysis. Among the demographic compositions, the gender distribution was roughly equal. Specifically, there were 2,644 males (49.8%) and 2,663 females (50.2%). The ages of the participants were between 14 and 20 years old, mainly concentrated in two groups: the majority were aged 15 (n = 2,523, 47.5%) and age 17 (n = 1,711, 32.2%). The distribution of academic grades showed an unbalanced state, as 60.3% of the students were in Grade 1, 39.7% were in Grade 3.

The results for mental health screening among adolescents had specific findings. It was found that 23.8% of adolescents (n = 1264) had a PHQ-9 score of ≥10, and 15.7% of adolescents (n = 834) had a GAD-7 score of ≥10. Achieving ≥10 on both questionnaires usually means the presence of moderate to severe clinical symptoms. Detailed demographic characteristics were shown in **Table 1**.

**Table 1:** Demographic characteristics of participants.

Variables		N	%
Age	14	17	0.3
	15	2523	47.5
	16	686	12.9
	17	1711	32.2
	18	354	6.7
	19	13	0.2
	20	3	0.1
Gender	Male	2644	49.8
	Female	2663	50.2
Academic level	Grade 1	3200	60.3
	Grade 3	2107	39.7
PHQ-9	≥ 10	1264	23.8
	< 10	4043	76.2
GAD-7	≥ 10	834	15.7
	< 10	4473	84.3

N = 5307. PHQ-9, Patient Health Questionnaire-9; GAD-7, Generalized Anxiety Disorder-7.

### 3.3 Descriptive statistics and correlation analysis

**Table 2** summarizes key variables and presents descriptive statistics and bivariate correlations. Aggression (M = 60.41, SD = 19.219) was significantly positively correlated with all other psychological variables in this analysis. Specifically, aggression was positively associated with internet addiction (r = 0.600, p < 0.01), somatization (r = 0.488, p < 0.01), depressive symptoms (r = 0.608, p < 0.01), and anxiety symptoms (r = 0.593, p < 0.01).

**Table 2:** Descriptive statistics and correlations among the variables.

Variables	Mean	SD	1	2	3	4	5
1. Agression	60.41	19.219	1				
2. Internet addiction	41.35	14.851	0.600**	1			
3. Somatization	5.76	5.628	0.488**	0.427**	1		
4. Depressive symptoms	6.67	5.198	0.608**	0.564**	0.671**	1	
5. Anxiety symptoms	5.21	4.735	0.593**	0.529**	0.642**	0.809**	1

N, 5307; SD, standard deviation. **p < 0.01.

The analysis also showed that internet addiction (M = 41.35, SD = 14.851) was correlated with somatization (r = 0.427), depressive symptoms (r = 0.564), and anxiety symptoms (r = 0.529). Furthermore, somatization (M = 5.76, SD = 5.628) showed a strong association with both depressive symptoms (r = 0.671) and anxiety symptoms (r = 0.642). These results provided the necessary foundation for the subsequent mediation analysis.

### 3.4 Multiple linear regression analysis

To examine the independent strength of association of the variables and check for multicollinearity, a multiple linear regression analysis was performed. As shown in **Table 3**, the Variance Inflation Factor (VIF) for all associated factors was well below the threshold of 10 (maximum VIF = 1.741), supporting that multicollinearity was not an issue in the regression models.

**Table 3:** Results from the multiple linear regression analysis examining the relationships among the variables.

	Depressive symptoms					Anxiety symptoms					
	Unstandardized		Standardized	t	p	Unstandardized		Standardized	t	P	VIF
	B	SE	β			B	SE	β			
Agression	0.070	0.003	0.257	21.955	<0.001	0.068	0.003	0.274	22.184	<0.001	1.741
Internet addiction	0.076	0.004	0.216	19.122	<0.001	0.058	0.004	0.181	15.173	<0.001	1.623
Somatization	0.419	0.010	0.453	43.760	<0.001	0.363	0.009	0.431	39.467	<0.001	1.362

N = 5307. SE, Standard Error.

For depressive symptoms, aggression (β = 0.257, t = 21.955, p < 0.001), internet addiction (β = 0.216, t = 19.122, p < 0.001), and somatization (β = 0.453, t = 43.760, p < 0.001) showed significant positive associations in the multivariable regression model. Similarly, Similarly, aggression (β = 0.274), internet addiction (β = 0.181), and somatization (β = 0.431) were significantly and positively associated with anxiety symptoms, all with p < 0.001.

### 3.5 Chain mediation analysis

**Figure 2:**
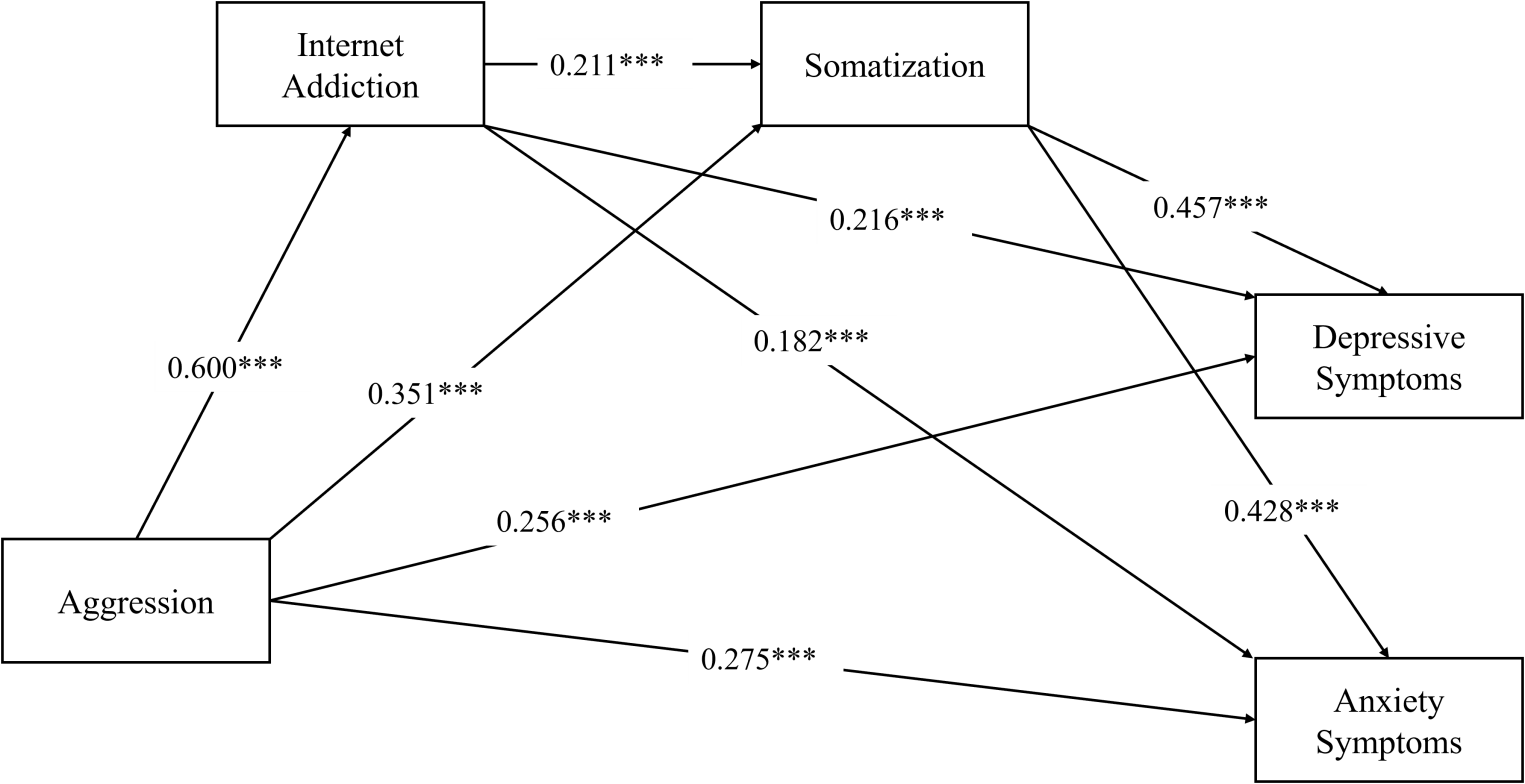
The chain mediating effect of internet addiction and somatization on the chain between aggression and mental health (depressive symptoms and anxiety symptoms). Coefficients are standardized β values. ***p <.001.

The hypothesized chain mediation model was tested using the PROCESS macro (Model 6). Regression analysis for each path was detailed in **Table 4**, and the standardized path coefficients were visualized in **Figure 2**.

**Table 4:** Regression analysis of the chain mediation model.

Variables	Step 1 Internet addiction (M_1_)		Step 2 Somatization (M_2_)		Step 3a Depressive symptoms (Y_1_)		Step 3b Anxiety symptoms (Y_2_)	
	β	t	β	t	β	t	β	t
Controls								
Gender	-0.009	-0.389	0.505***	22.488	-0.017	-0.908	0.015	0.778
Age	0.008	0.787	0.066***	6.202	-0.006	-0.673	0.008	0.862
Independent Var.								
Aggression (X)	0.600***	54.507	0.351***	25.000	0.256***	21.835	0.275***	22.174
Mediators								
Internet addiction (M_1_)			0.211***	15.024	0.216***	19.020	0.182***	15.193
Somatization (M_2_)					0.457***	42.004	0.428***	37.312
R^2^	0.360		0.334		0.582		0.535	
F	994.927***		663.498***		1478.503***		1221.225***	

N = 5307. β represents standardized regression coefficients. Gender was dummy coded (1 = Male, 2 = Female). Step 1 and Step 2 are identical for both models. ***p <.001.

In the first step, aggression was found to be substantially and positively associated with internet addiction (β = 0.600, t = 54.507, p < 0.001). In the second step, both aggression (β = 0.351, t = 25.000, p < 0.001) and internet addiction (β = 0.211, t = 15.024, p < 0.001) were significantly associated with somatization.

Finally, for the outcome variables, the total model explained 58.2% of the variance in depressive symptoms (F = 1478.503, p < 0.001) and 53.5% of the variance in anxiety symptoms (F = 1221.225, p < 0.001). Specifically, aggression (β = 0.256), internet addiction (β = 0.216), and somatization (β = 0.457) were all independently and positively associated with depressive symptoms. For anxiety, the paths from aggression (β = 0.275), internet addiction (β = 0.182), and somatization (β = 0.428) were also significant.

### 3.6 Mediation effects and bootstrap results

The indirect effects were further evaluated using 5,000 bootstrap resamples. As indicated in **Table 5**, the total indirect effect of aggression on depressive symptoms was 0.347 (95% CI [0.324, 0.370]), and on anxiety symptoms was 0.313 (95% CI [0.291, 0.335]). Since none of the 95% confidence intervals included zero, all indirect pathways were deemed statistically significant. To quantify the practical effect size of the mediation, the proportion of mediation was calculated. The total indirect effect accounted for 57.5% (0.347/0.603) of the total effect of aggression on depressive symptoms, and 53.2% (0.313/0.588) of the total effect on anxiety symptoms. These proportions indicate that internet addiction and somatization may account for a substantial part of the statistical association between aggression and internalizing symptoms within the proposed cross-sectional mediation model.

**Table 5:** Bootstrap analysis of indirect effects for depressive symptoms and anxiety symptoms.

Effects	Outcome: depressive symptoms (Y_1_)			Outcome: anxiety symptoms (Y_2_)		
	Effect	Boot SE	95% CI	Effect	Boot SE	95% CI
**Total effect**	0.603	0.011	[0.582, 0.625]	0.588	0.011	[0.566, 0.609]
**Direct effect**	0.256	0.012	[0.233, 0.279]	0.275	0.012	[0.250, 0.299]
**Total indirect effect**	0.347	0.012	[0.324, 0.370]	0.313	0.011	[0.291, 0.335]
Specific indirect paths						
Agg → IA → Y	0.129	0.009	[0.112, 0.147]	0.109	0.009	[0.092, 0.126]
Agg → Som → Y	0.160	0.009	[0.142, 0.179]	0.150	0.009	[0.133, 0.168]
Agg → IA → Som → Y	0.058	0.005	[0.048, 0.068]	0.054	0.005	[0.045, 0.063]

Bootstrap sample = 5,000. Boot SE, Bootstrap Standard Error; CI, Confidence Interval; Agg, Aggression; IA, Internet Addiction; Som, Somatization. All indirect paths are significant (CIs do not include 0).

The indirect effect was composed of three specific paths:

1. The mediating role of internet addiction: The path aggression → internet addiction → Y was significant for both depressive symptoms (β = 0.129, 95% CI [0.112, 0.147]) and anxiety symptoms (effect = 0.109, 95% CI [0.092, 0.126]).
2. The mediating role of somatization: The path aggression → somatization → Y was significant for depressive symptoms (β = 0.160, 95% CI [0.142, 0.179]) and anxiety symptoms (β = 0.150, 95% CI [0.133, 0.168]).
3. The chain mediating role of internet addiction and somatization: The sequential path aggression → internet addiction → somatization → Y was also significant for depressive symptoms (β = 0.058, 95% CI [0.048, 0.068]) and anxiety symptoms (β = 0.054, 95% CI [0.045, 0.063]).

To summarize the findings, both internet addiction and somatization functioned in a dual capacity within the investigated relationship: they acted as individual, stand-alone mediators linking aggression to mental health and also operated in a sequential path, forming a chain of mediation, with internet addiction preceding somatization, through which aggression exerted its influence on the development of internalizing psychological symptoms.

## 4 Discussion

Based on a sample of 5,307 Chinese adolescents, this study examined a theoretically derived chain mediation model linking aggression with depressive and anxiety symptoms through internet addiction and somatization. The findings suggest that aggression was associated with both emotional outcomes directly and indirectly through behavioral and body related mediators. Importantly, because the study used a cross-sectional design, these findings should be interpreted as statistical associations rather than evidence of causal or temporal ordering. The proposed sequence from aggression to internet addiction, somatization, and emotional distress is theoretically plausible, but it represents only one possible configuration among several potential developmental pathways. In our samples, 23.8% adolescents had moderate to severe depressive symptoms, and 15.7% adolescents had moderate to severe anxiety symptoms. A key comparative finding emerged from the analysis of the two outcome variables. Although the established model accounted for a substantial proportion of variance in both depressive and anxiety symptoms, the explanatory power was higher for depressive symptoms than for anxiety symptoms. This difference suggests distinct underlying mechanisms. Specifically, somatization, as a mediating factor, showed a stronger association with depressive symptoms than with anxiety symptoms in this chain mediation model. The significance of these findings can help clarify the heterogeneous mechanisms underlying the comorbidity of externalizing problems (such as aggression) and internalizing problems (such as depressive and anxiety symptoms). Furthermore, the generated empirical evidence provides key support for the development and implementation of intervention models, especially those for school environments.

### 4.1 Demographic vulnerabilities: the influence of gender and grade

Before conducting a detailed inspection of the core mediation path, an important analysis step needs to be carried out. In particular, the results of our chain mediation analysis showed that both gender and age were significant associated factors of somatization, and the direction of the relationships suggested that female and older adolescents reported more somatic symptoms than their male and younger counterparts.

This difference between genders, with females reporting more complaints of their somatic symptoms, is consistent with the existing literature. Research suggests that adolescent girls have greater interoceptive awareness, and their social structure is in a way to express psychological distress through physical symptoms as opposed to behavioral patterns in boys (47, 48). If we interpret this as part of our “trait-behavior” model, one possible difference is further apparent: while male adolescents might more often demonstrate direct behavior manifestations of aggressive traits, female adolescents show a higher physiological burden as a consequence of stress due to the same underlying aggressive predisposition.

The finding that age growth is associated with higher somatization scores is quite eye-catching. This view reflects the unique high school ecology in China. From the first year of high school to the year before the college entrance examination in the third year of high school, academic pressure gradually increases, which may accelerate physical wear and tear, leading to symptoms such as headaches and fatigue. This grade related effect shows that the path from aggression to somatization is not invariable. Instead, teenagers are experiencing and having to cope with increasingly escalating pressure in the Chinese education system, and this trend is becoming more intense. Therefore, this finding highlights the actual need for interventions targeting specific grades, as senior students may need more intensive and targeted help with physical symptom management than junior students due to the greater accumulation of pressure.

### 4.2 The behavioral pathway: aggression driving digital escapism

One important finding is the strong positive association between aggression and internet addiction, identifying hostile personality traits as *a priori*, and before the onset of maladaptive coping strategies that occur in digital settings. This is consistent with the Social Compensation Hypothesis: Young people with aggressive traits are likely to be rejected by peers in offline social situations and may lack some social skills, thus, they often turn to online environments to achieve their desired goals (e.g., dominance or emotional release) (20, 21). The conclusion obtained from our analysis is also very valid in a second, methodologically distinct study done by Gan et al. In their machine learning analysis study, with a similar sample of Chinese adolescents, they identified “hostility” and “psychoticism” as top-performing associated factors for adolescent internet addiction and found an Extreme Gradient Boosting model to effectively focus on certain psychological traits, such as impulsive and hostile traits, that are potential early indicators for internet addiction (27). Their numerical results provide a strong proof of principle, consistent with and supportive with the results of our statistical models.

However, there is a theoretical tension when we directly compare the results of this study with those of Zhu et al. Their research results show that mobile phone addiction (MPA) is the initial factor in the development of anxiety and depression which in turn triggers aggressive behavior—a process they attribute to the withdrawal symptoms and emotional dysregulation caused by the addictive behavior (49). Using a longitudinal cross-lagged panel design, Yi and Li also found that depressive symptoms cause internet addiction, a result for a “self-medication” interpretation—where addictive behavior is seen as a consequence of pre-existing psychopathology rather than a cause (18).

The apparent discrepancy in causal direction—whether aggression leads to internet addiction or vice versa—may be reconciled by distinguishing between aggression as a stable trait versus a reactive state. Our measurement method was BPAQ, which was meant to be able to measure aggression as the stable personality trait, including hostile and trait anger (36). From a developmental psychopathology point of view, this is logical: these long-lasting traits may be formed before specific habits, such as internet usage (50). Aggressive individuals tend to have a hostile attribution bias cognitive pattern which leading them to perceive offline social cues as threats—this negative perception pushes them toward virtual environments for safety, where their subsequent addiction serves as a maladaptive coping mechanism (51).

In contrast, the studies by Zhu et al. and Yi & Li may have been more indicative of the severity of symptoms of the pathological process. Once established, addiction can lead to sleep problems and social isolation, creating a psychological state that lowers the threshold for aggression. These consequences may also aggravate the previous depressive state.

Therefore, a comprehensive model must recognize that this relationship is long-term and bidirectional. It is highly plausible that internalizing symptoms themselves contribute to increased aggression or problematic internet use as maladaptive coping mechanisms, creating a vicious cycle. Furthermore, these dynamics must be interpreted within the unique cultural context of Chinese adolescents. In societies where openly expressing psychological distress can sometimes carry stigma, emotional pain is frequently channeled into culturally acceptable somatic complaints. Coupled with the intense, highly competitive academic pressure characteristic of the Chinese educational system, digital media often serves as a primary, yet hazardous, refuge for stress relief.

However, our current data highlights a specific insight: in the development of adolescent psychological disease, the trait driven path (aggression to internet addiction) seems to have a dominant and initiating role.

### 4.3 The physiological bridge: somatization as the key factor

Our main result is that somatization provides a link between dysfunctional behaviors and the subsequent emotional distress. In a previous study, Wang et al. focused on cognitive deficits and found that inhibitory control plays a mediating role between victimization experience and social network addiction (52). Our study extends this line of inquiry by taking another step beyond cognitive areas and exploring the physiological cost of such harmful behaviors. The analysis results show that internet addiction is associated with the aggravation of somatization, and the elevated somatization in turn acts as a proximal factor that subsequently intensifies the severity of both depressive symptoms and anxiety symptoms.

This finding incorporates the biopsychosocial model into the context of the digital age. Pathological internet use is not only a form of cognitive preoccupation, but also accompanied by physiological changes, such as long-term lack of exercise, severe circadian rhythm disruption, and persistent sensory overload (24–26). These enduring stressors can keep the hypothalamus-pituitary-adrenal (HPA) axis and the sympathetic nervous system in a chronically activated state. Ultimately, this physiological hyperactivation manifests externally in the form of symptoms represented by the various somatic complaints assessed in our study, such as headaches, stomach discomfort, and chronic fatigue.

Comparing this finding with the research by Bekhuis et al. can provide further insights. Bekhuis et al.’s network analysis of depression and anxiety revealed that specific somatic complaints (e.g., fatigue, abnormal cardiopulmonary function) act as critical connectors between somatic and psychiatric symptoms, and that clusters such as cardiopulmonary or gastrointestinal symptoms can be associated with a worse prognostic outcome for depression (53). The research we carried out extends this to the adolescent group, suggesting that internet addiction is a modern risk related behavioral factor. This factor may be related to specific somatic symptom clusters. Once these symptom clusters are formed, they will accelerate the process of full internalization disorders. So, we argue that the bodies of adolescents show early signs of distress before obvious psychological distress appears. This makes somatization a possible early warning signal of mental health decline related to excessive internet use.

Although our model specified internet addiction as preceding somatization based on the biopsychosocial framework and the physiological consequences of excessive internet use, this ordering should be interpreted as theoretical rather than causal. Alternative explanations are also plausible. Adolescents with pre-existing somatic complaints, such as fatigue, headaches, or gastrointestinal discomfort, may reduce offline activities and rely more heavily on internet use for distraction, social connection, or emotional relief. In this case, somatization may contribute to problematic internet use rather than merely follow it. Moreover, internet addiction and somatization may reinforce each other over time: excessive internet use may worsen sleep, physical inactivity, and bodily discomfort, whereas persistent somatic symptoms may further increase avoidance of offline activities and reliance on digital environments. Therefore, the present model should be viewed as one theoretically plausible cross-sectional configuration. Future longitudinal and cross lagged studies are needed to compare the proposed sequence with alternative models, including somatization to internet addiction and reciprocal pathways.

### 4.4 Depression and anxiety: specific associations or general distress?

In our study, the established chain mediation model showed that the total variance proportion of depressive symptoms was greater than that of anxiety symptoms. Furthermore, the associated path strengths from the intermediary variables to each result were not equivalent. The standard path coefficient of somatization to depressive symptoms was observed to be stronger than that of somatization to anxiety symptoms.

The impact of this difference may be explained by the convergence of the phenomenology of somatization and the clinical manifestations of depression. Depression is usually characterized by “sick behavior” symptoms, such as pronounced lethargy, heightened sensitivity to pain, and various vegetative symptoms. These symptoms are close consistent with the physical burden quantified by the PHQ-15.

Previous studies, including the longitudinal research by Li et al., have indicated that within Chinese adolescent samples, depression often demonstrates greater temporal stability and possesses stronger strength of association for subsequent negative outcomes when compared to anxiety (9). Zhu et al.’s research also shows that anxiety is often a prerequisite leading to the onset of depression (49).

In our model, the somatic symptoms resulting from internet addiction can be associated with physical exhaustion or a deep sense of fatigue, which may mimic one of the core symptoms of depression—profound exhaustion. This mimicry could thereby strengthen the connection between somatization and depressive symptoms. Conversely, anxiety is primarily characterized by a state of hyperarousal and vigilance. This anxious state may be more directly driven by specific psychological content encountered during internet use, such as the fear of missing out (FOMO) or engagement in unfavorable social comparisons. These cognitive-emotional factors are different from the direct physical effects brought about by addiction (such as chronic fatigue), which are a primary pathway leading to somatization.

A further issue concerns the substantial overlap between depressive and anxiety symptoms. In the present sample, PHQ-9 and GAD-7 scores were highly correlated, suggesting that the two outcomes may partly reflect a broader general distress factor rather than entirely distinct clinical constructs. Therefore, the stronger association between somatization and depressive symptoms should be interpreted cautiously. It may indicate a relatively closer link between somatic burden and depressive manifestations, but it may also reflect the shared negative affectivity underlying both depression and anxiety. The present study compared depressive and anxiety symptoms as two commonly used dimensional outcomes; however, it did not formally model a latent general distress factor. Future studies could use bifactor models, latent variable modeling, or transdiagnostic approaches to distinguish symptom specific pathways from general internalizing distress.

### 4.5 Clinical implications: implementing the “HSHC” model

The pathways among psychological traits, external behaviors, and physiological responses are rather complex. Such complexity requires a systematic approach to intervention design and implementation. The empirical results of our research strongly support a particular service framework called “Hospital-School-Home-Community” (HSHC) integrated mental health service model comprehensively described and analyzed by Hong et al. (54).

Hong et al. presented a “care pathways” blueprint for screening over 330,000 students in Chongqing, attributing its key success to the integration of universal school screening with specialized hospital care (54). Our study employed samples from the same region, validated the scientific necessity of specific components within the model, and provided recommendations for its refinement.

Enhanced Screening Protocols: Hong et al. employed established instruments, specifically the PHQ-9 and the GAD-7, for their screening purposes. The results of our analysis are associated with an extension of such a screening program. In our model, somatization is a potential early indicator of clinical depressive symptoms. Early detection of students with unexplained physical discomforts can enable the adoption of targeted intervention measures to prevent potential comprehensive mental disorders. Therefore, we suggest integrating somatization assessment (for example, using PHQ-15) into the screening framework.

Gatekeeper Training: Hong et al. proposed that key personnel such as teachers and parents, who act as gatekeepers, should be trained to improve the recognition of mental health risks. However, our research found that the scope of this training needs to be expanded. Gatekeepers should be trained not only to detect psychotic symptoms but also to acquire practical methods for behavior management. Specifically, we recommend incorporating targeted interventions such as Cognitive Behavioral Therapy for Internet Addiction (CBT-IA) into school counseling frameworks to help adolescents identify aggressive triggers and regulate their digital behaviors. Furthermore, somatic symptom monitoring should be paired with psychoeducation and body-oriented therapies, such as Mindfulness-Based Stress Reduction (MBSR). These programs can be tailored for aggressive adolescents to help them physically de-escalate and manage bodily distress before it compounds into severe depressive or anxiety disorders. Consequently, carefully designed interventions need to channel potential aggressive energy towards prosocial, structured offline activities. Adopting a simplistic strategy of internet restriction may prove counterproductive because research suggests it can trigger a maladaptive cycle of withdrawal and subsequent aggression (49).

Medical-Educational Collaboration: In the HSHC model, the green channel mechanism for rapidly transferring symptomatic students to hospitals for examination and treatment is a crucial component. Our collected data revealed that 23.8% of the students we surveyed exhibited depressive symptoms reaching a moderate level of severity. This prevalence is driven by complex interactions (such as somatization), which exceed the management capacity of school counseling resources acting in isolation. Therefore, structured cooperation with professional psychiatric centers is crucial (a core part of HSHC). This cooperation guarantees the comprehensive management of students’ physical, biological, psychological and emotional problems.

### 4.6 Limitations and future directions

There are several limitations in this study. First, the cross-sectional nature of our data inherently restricts the ability to draw definitive causal inferences. Although our chain mediation model implies a temporal sequence from trait aggression to emotional distress, the current design only allows for testing statistical mediation, rather than true causality or directionality. Previous longitudinal studies have also offered a completely different perspective. For instance, Li and colleagues demonstrated that the relationship between aggression and mental health outcomes may be bidirectional over a three-year observation period (9). Thus, pre-existing depressive symptoms may also exacerbate aggressive tendencies, forming an interactive feedback loop over time. Future longitudinal or experimental designs are required to validate the causal trajectory.

Second, while the sample size is large, only Grade 1 and Grade 3 high school students were included. The absence of students from Grade 2 makes a gap in the development timeline of high school students. Adolescence is usually understood as a life period of rapid psychological flux. By missing data from this intermediate year, our study is prevented from capturing the potential progression of key variables.

Third, the tool used in this study is based on self-report. Although Harman’s single factor test suggested that a single common factor did not dominate the variance, this procedure is only a preliminary diagnostic method and cannot fully exclude common method bias. Shared response styles, negative affectivity, social desirability, and recall bias may have inflated the observed associations among aggression, internet addiction, somatization, depressive symptoms, and anxiety symptoms. Future studies should incorporate multi informant assessments, clinical interviews, teacher or parent reports, behavioral indicators of internet use, and objective physiological measures.

Fourth, although the investigation focused on the construct of somatization as a key mediator, the assessment was limited to self-reported symptoms and there was no direct measurement of specific biomarkers that could provide a mechanistic explanation for the observed link between somatization and mental health. Examples of such biomarkers include cortisol levels as an indicator of HPA-axis activity or various inflammatory markers. Future research should carry out work to integrate these objective biomarkers. This integration can verify the rationality of physiological mechanisms in our theoretical model.

Fifth, to comply with school administrative guidelines regarding sensitive topics for adolescents, the item assessing sexual activity was excluded from the PHQ-15. While this exclusion is methodologically understandable within this specific cultural and educational context, we acknowledge it as a limitation, as it may affect the direct comparability of our somatic symptom severity findings with clinical studies utilizing the complete scale.

Sixth, only gender and age were controlled in the present model. Several important confounders were not assessed, including socioeconomic status, family environment, academic stress, peer relationships, sleep quality, physical activity, and prior mental health history. These factors may influence aggression, internet addiction, somatic complaints, and emotional symptoms simultaneously. For example, academic stress may increase both internet use as an avoidance strategy and somatic complaints such as fatigue or headaches. Therefore, the observed indirect effects may partly reflect unmeasured contextual or clinical influences. Future studies should include a broader set of covariates and examine whether the proposed mediation pattern remains robust after adjustment for these factors.

Finally, while our chain mediation model controlled for gender as a covariate, we did not explore potential moderated mediation effects. Given the established epidemiological evidence that aggression and internet addiction often exhibit distinct gender-specific trajectories (55), future research should utilize multi-group analysis to determine whether the magnitude of these mediational pathways differs significantly between male and female adolescents.

## 5 Conclusion

In summary, our results provide a clear indication of a pathway from aggression to emotional distress. Two key factors play sequential mediating roles: first, internet addiction behavior, followed by somatic symptoms. Specifically, we uncovered a strong connection between internet addiction and psychological health issues. This finding highlights a critical need for modern mental health practice: good psychological treatments must take the physical state into consideration in order to offer a high level of care. We support integrated models, such as the “Hospital-School-Home-Community” model, in which behavioral risks, somatic complaints, and psychological symptoms are monitored simultaneously and vulnerable children are recognized and supported holistically.

## Data Availability

The raw data supporting the conclusions of this article will be made available by the authors, without undue reservation.
